# Sex differences in health status, healthcare utilization, and costs among individuals with elevated blood pressure: the LARK study from Western Kenya

**DOI:** 10.1186/s12889-021-10995-3

**Published:** 2021-05-19

**Authors:** Neha Sikka, Allison DeLong, Jemima Kamano, Sylvester Kimaiyo, Vitalis Orango, Josephine Andesia, Valentin Fuster, Joseph Hogan, Rajesh Vedanthan

**Affiliations:** 1 Icahn School of Medicine at Mount Sinai, New York, USA; 2 Center for Statistical Sciences, School of Public Health, Brown University, Providence, RI USA; 3 Department of Medicine, Moi University College of Health Sciences, Eldoret, Kenya; 4 Academic Model Providing Access to Healthcare (AMPATH), Eldoret, Kenya; 5 Department of Biostatistics, Brown University School of Public Health, Providence, RI USA; 6 Department of Population Health, NYU Grossman School of Medicine, New York, NY USA

**Keywords:** Hypertension, Sex differences, Healthcare utilization, Healthcare costs

## Abstract

**Background:**

Elevated blood pressure is the leading risk factor for global mortality. While it is known that there exist differences between men and women with respect to socioeconomic status, self-reported health, and healthcare utilization, there are few published studies from Africa. This study therefore aims to characterize differences in self-reported health status, healthcare utilization, and costs between men and women with elevated blood pressure in Kenya.

**Methods:**

Data from 1447 participants enrolled in the LARK Hypertension study in western Kenya were analyzed. Latent class analysis based on five dependent variables was performed to describe patterns of healthcare utilization and costs in the study population. Regression analysis was then performed to describe the relationship between different demographics and each outcome.

**Results:**

Women in our study had higher rates of unemployment (28% vs 12%), were more likely to report lower monthly earnings (72% vs 51%), and had more outpatient visits (39% vs 28%) and pharmacy prescriptions (42% vs 30%). Women were also more likely to report lower quality-of-life and functional health status, including pain, mobility, self-care, and ability to perform usual activities. Three patterns of healthcare utilization were described: (1) individuals with low healthcare utilization, (2) individuals who utilized care and paid high out-of-pocket costs, and (3) individuals who utilized care but had lower out-of-pocket costs. Women and those with health insurance were more likely to be in the high-cost utilizer group.

**Conclusions:**

Men and women with elevated blood pressure in Kenya have different health care utilization behaviors, cost and economic burdens, and self-perceived health status. Awareness of these sex differences can help inform targeted interventions in these populations.

**Supplementary Information:**

The online version contains supplementary material available at 10.1186/s12889-021-10995-3.

## Background

Elevated blood pressure is the leading global risk factor for mortality and the most common cardiovascular condition in the world [1]. Despite 80% of all cardiovascular-related deaths occurring in low- and middle-income countries, health care utilization in these populations remains low [2, 3]. Differences in healthcare utilization by sex have been widely reported, with higher use by women [4–6]. This higher utilization is furthermore associated with increased healthcare costs [6, 7].

**Fig. 1:**
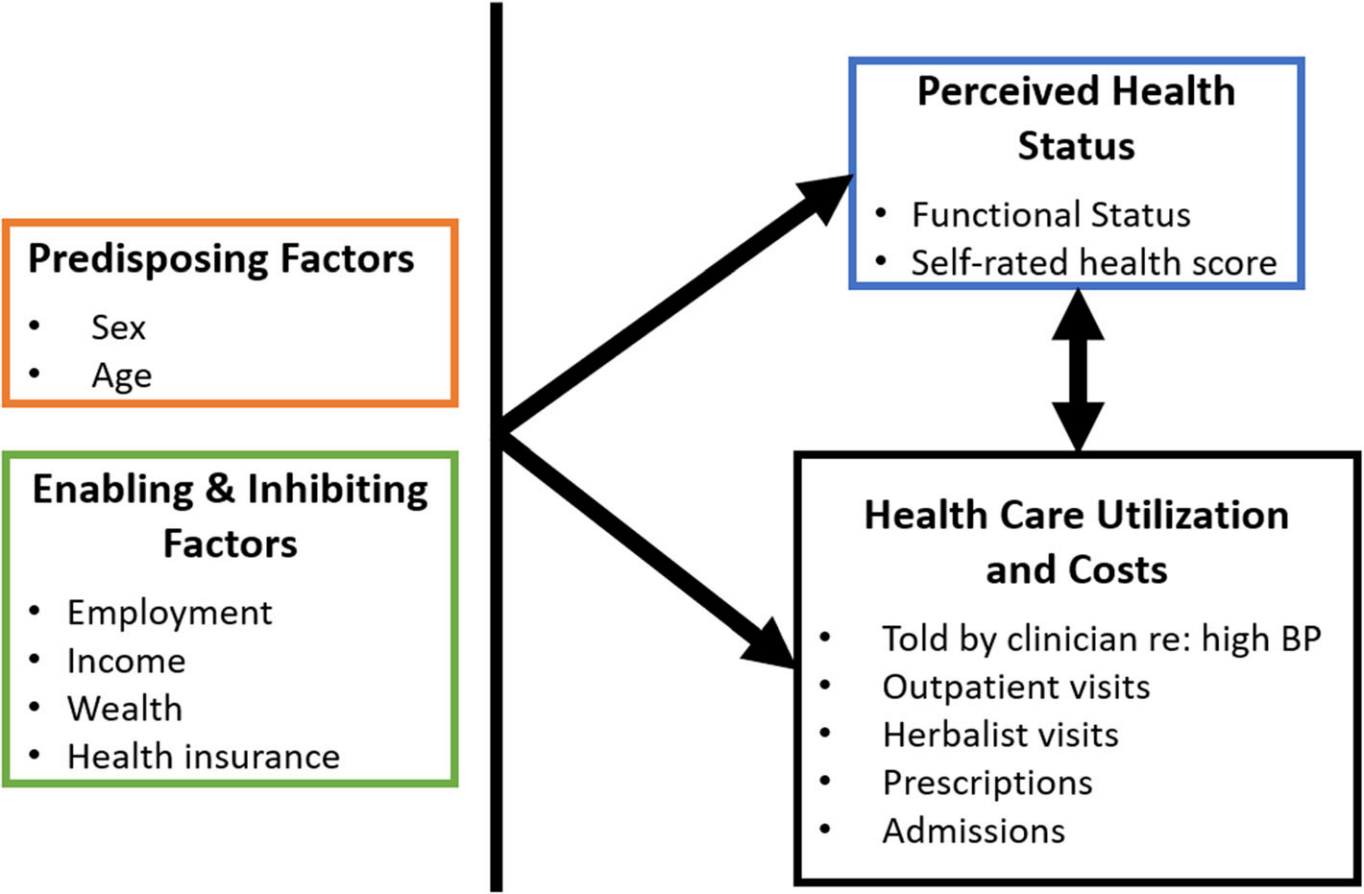
Factors that may impact healthcare utilization. Potential variables examined in this study, categorized as predisposing factors, enabling/inhibiting factors, and perceived health status

Healthcare utilization is influenced by three groups of factors: “predisposing factors” which include sex along with age, educational level, marital status, and trust level in healthcare influence; “enabling/inhibiting factors” such as medical insurance, wealth, and availability of medical care; and need for care (Fig. 1) [8, 9]. Emerging literature has supported sex differences in relation to many of these individual characteristics. For instance, the 2007–2016 NHANES survey of US civilian populations found higher awareness, treatment, and blood pressure control rates in women age less than 65 years with hypertension of all races [10], despite general lower rates of employment and lower income compared to men of the same status [11, 12]. However, characterizations of the differences in health care utilization, cost, and associated factors between men and women with elevated blood pressure is limited in African populations.

We therefore sought to describe the patterns and costs of health care utilization of men and women with elevated blood pressure in western Kenya along with characteristics that may affect these patterns. The LARK Hypertension study is a cluster randomized controlled trial that demonstrated that community health workers, equipped with behavioral communication strategies and smartphone decision-support tools, can increase linkage to hypertension care and yield modestly improved but not statistically significant blood pressure reduction among individuals with hypertension in rural Western Kenya [13]. We present here an analysis of baseline data from the trial, focusing on sex differences in self-reported health status, healthcare utilization, and costs in this population.

## Methods

### Study setting and participants

The LARK study was conducted within the infrastructure of the Academic Model Providing Access to Healthcare Partnership (AMPATH) in Kenya [14, 15]. It was conducted within two administrative divisions in rural western Kenya: Kosirai and Turbo. From April 2014 to December 2016, adult individuals who met the study inclusion criterion of elevated blood pressure (defined as systolic blood pressure (SBP) ≥ 140 mg/dL or diastolic blood pressure (DBP) ≥ 90 mg/dL) were invited and consented into the LARK study. Individuals without elevated blood pressure, those who did not provide informed consent, or those who were critically ill were excluded. Individuals who were actively engaged in hypertension care were also excluded. Overall, the LARK study enrolled 1460 participants. Full data for the present analysis were available for 1447 participants. Written informed consent was obtained from all participants. Detailed study procedures have previously been fully reported [14].

### Survey

The baseline questionnaire collected data about employment status, field of employment, and reason for unemployment when relevant. Monthly income was asked in 5000 to 10,000 Kenyan shilling (KS) increments (roughly equivalent to $50 - $100 increments). Participants were asked whether they had health insurance, including the Kenyan National Health Insurance Fund (NHIF). All participants were asked if they had been told about their elevated blood pressure by a healthcare worker or doctor in the past 12 months. Rates of health care utilization were determined by questions on numbers of admissions to a hospital (in-patient) over the past 12 months, and number of visits to an outpatient medical provider, herbalist, or spiritual provider over the past 3 months. Participants with any of the previous visits were asked for their out-of-pocket costs for the visit. Additionally, participants were asked about medications they were prescribed and associated cost. To approximate quality-of-life status, participants were asked about mobility, self-care, ability to perform usual activities, pain, and anxiety/depression. Participants were also asked to score their health status on a scale of 0–100, with 0 being “the worst health you can imagine” and 100 being “the best health you can imagine” [16]. All items in the questionnaire were ascertained by self-report. Sex (“male” or “female”) of each participant was recorded by the clinician in the clinical encounter form. All baseline questionnaire data were collected prior to any LARK study intervention.

### Data analysis

Demographic, socioeconomic and health status variables and self-reported measures healthcare utilization over the past 3 or 12 months were summarized overall and separately for men and women. Categorical measures were expressed using counts, and percentages and continuous measures using median and interquartile range (IQR). Data were analyzed using R version 3.6.1 [17].

### Health utilization and costs

Latent class regression analysis (LCA) was used to describe patterns of healthcare utilization and costs in our population [18]. A latent class distribution was assumed to describe the joint distribution of five manifest (dependent) variables: one binary variable (whether they had been told by a health worker or doctor they had high blood pressure in past 12 months (with the 27 observations with missing values omitted)); and four multinomial variables, each with three mutually exclusive classes (1)*:* cost of hospital admissions in the past 12 months (no admission, less than 5000 KS (~ 50 USD), more than 5000 KS) (2)*;* cost of outpatient visit in the past 3 months (no outpatient visit, less than 200 KS (~ 2 USD), more than 200 KS) (3)*;* cost of visit to a herbal medicine or spiritual healer in the past 3 months (no herbal medicine/spiritual healer visit, less than 200 KS, more than 200 KS); and (4) cost of prescription medication filled in the past 3 months (no prescriptions, less than 200 KS, more than 200 KS). Cut-offs for cost variables were based on the data including median cost values and burden based on income. Since “being told by a healthcare worker about high blood pressure status” may not fully reflect healthcare utilization of all participants, we performed a secondary LCA without this manifest variable.

The patterns of health utilization and costs for each latent class were described and an informative label was assigned to each class, anticipating finding LCA groups pertaining to low, medium, and high health care use and costs. The probability of belonging in each latent class was captured for each participant. For descriptive summaries, participants were assigned to the class with the highest probability.

The latent class regression analysis allowed the dependent manifest variables to be modeled as a function of covariates. We allowed latent class membership probability to be dependent on sex, age group (< 50, 50–64, > = 65), health insurance status, employment and income status as a 3-level variable (no job, monthly earnings <5000KS, and earnings > = 5000KS), and community unit [19]. Observations with missing data (*n* = 108 (7.5%)) were omitted from this analysis. Using the largest latent class as the reference, we generated relative risk ratios of latent class membership for the other classes by sex, age, insurance, and employment/income status. LCA models were fit using the poLCA R package [19]. The Akaike information criterion (AIC) was used for model selection [17].

### Utilization and self-reported health

Self-reported health status was summarized by latent class assignment. To examine our primary hypothesis that there were sex differences in health status, utilization and costs, we regressed the self-reported health measures on latent class membership probability and gender, adjusting for demographics. Specifically, for each of the 6 health status measures (5 binomial and 1 continuous), a mixed effects regression model with a random effect for community unit was used to examine the relationship between each health status as the dependent variable and the probability of latent class membership (using the largest group as the reference) and sex. All models included covariates for age group, health insurance status, and employment and income status. For the continuous health score, the effects measured the difference in health status. For the binomial symptom measures (pain, anxiety and depression, mobility, self-care, and ability to complete usual activities) we used logistic mixed effects models and compared having any symptoms to no symptoms using the odds ratio (OR).

## Results

### Demographics and self-rated health

Of the 1447 participants, 58% were women. Women were more likely to be unemployed (Table 1). Of those not working, 40% of women and 63% of men indicated they were retired or too old. Excluding this, the top reason for not working reported by women was that they were caring for family, whereas for men, the next most cited reason was inability to find work. Among those with formal employment, women were more likely to report earning less than 5000 KS (~ 50 USD) per month. A large proportion of the study population was not enrolled in health insurance of any type, with only 13% of women and 17% of men indicating enrollment in Kenyan NHIF. Women reported worse self-reported quality-of-life than men, with more women reporting issues with mobility, ability to perform usual activities, pain, anxiety and depression, and lower overall median health score compared to men.

**Table 1:** Summary of participant demographic characteristics and self-rated health by sex

Category	Total ***N*** = 1447	Female ***N*** = 838	Male ***N*** = 609
Age (years)	55.0 (42.0, 66.0)	54.0 (42.0, 65.0)	56.0 (40.0, 67.0)
Employment			
No Job	304 (21)	231 (28)	73 (12)
Farmer	728 (50)	431 (51)	297 (49)
Business Person	185 (13)	104 (12)	81 (13)
Public Sector Employee	54 (4)	17 (2)	37 (6)
Student	4 (0)	3 (0)	1 (0)
Other	150 (10)	43 (5)	107 (18)
Missing	22 (2)	9 (1)	13 (2)
Reason for Not Working			
Retired or too old	138 (45)	92 (40)	46 (63)
Caring for Family	68 (22)	65 (28)	3 (4)
Could not find or get work	41 (13)	31 (13)	10 (14)
Illness or Disability	38 (12)	32 (14)	6 (8)
In School	6 (2)	1 (0)	5 (7)
Temporary Gap in Employment	5 (2)	4 (2)	1 (1)
Other	8 (3)	6 (3)	2 (3)
Monthly Earnings Among Working (KS)			
< 5000	712 (62)	438 (72)	274 (51)
≥ 5000 & < 10,000	198 (17)	78 (13)	120 (22)
≥ 10,000 & < 20,000	78 (7)	23 (4)	55 (10)
≥ 20,000 & < 30,000	36 (3)	13 (2)	23 (4)
≥ 30,000	28 (2)	8 (1)	20 (4)
Missing	91 (8)	47 (8)	44 (8)
Have NHIF			
Yes	213 (15)	110 (13)	103 (17)
No	1205 (83)	712 (85)	493 (81)
Missing	29 (2)	16 (2)	13 (2)
How would you describe your pain?			
No pain	721 (50)	349 (42)	372 (61)
Moderate pain	677 (47)	459 (55)	218 (36)
Extreme pain	28 (2)	22 (3)	6 (1)
Missing	21 (1)	8 (1)	13 (2)
How would you describe your anxiety or depression?			
Not anxious	635 (44)	318 (38)	317 (52)
Moderately anxious	671 (46)	428 (51)	243 (40)
Extremely anxious or depressed	120 (8)	84 (10)	36 (6)
Missing	21 (1)	8 (1)	13 (2)
How would you describe your mobility?			
No problems in walking	972 (67)	517 (62)	455 (75)
Some problems in walking	448 (31)	308 (37)	140 (23)
Confined to bed	3 (0)	2 (0)	1 (0)
Missing	24 (2)	11 (1)	13 (2)
How would you describe your self-care?			
No problems with self-care	1321 (91)	759 (91)	562 (92)
Some problems washing or dressing	98 (7)	66 (8)	32 (5)
Unable to wash or dress myself	6 (0)	4 (0)	2 (0)
Missing	22 (2)	9 (1)	13 (2)
How would you describe your usual activities?			
No problems with usual activity	1148 (79)	632 (75)	516 (85)
Some problems performing usual activity	257 (18)	185 (22)	72 (12)
Unable to perform usual activity	18 (1)	11 (1)	7 (1)
Missing	24 (2)	10 (1)	14 (2)
How is your health today, 0–100?			
	70.0 (60.0, 80.0)	70.0 (60.0, 80.0)	75.0 (65.0, 82.5)
Missing	22	8	14

All monetary values in are Kenyan Shillings. Continuous variables are presented as “median (IQR)” and categorical variables as N (%). Percentages are by column

### Healthcare utilization and associated costs

Women reported higher rates of having been told about their elevated blood pressure within the past 12 months, attendance at an outpatient medical visit, and taking prescription medication (Table 2). Women and men had similar low rates of hospital admissions over the previous 12 months, with less than 1 % of the participants having multiple admissions. Men and women also had similar rates of visits to herbalists or spiritual leaders, with almost one-fifth of participants seeking these alternative care sources. A higher proportion of women had no costs for their outpatient visits, though a lower proportion of women paid ≤200 KS for their outpatient visit compared to men. Similarly, a higher proportion of women paid no cost for herbalist visits, but a lower proportion of women paid ≤200 KS for their herbalist visit.

**Table 2:** Healthcare utilization and cost by sex

Category	Value	Total *N* = 1447	Female *N* = 838	Male *N* = 609
Told have high BP in past 12 months?				
	Yes	585 (40)	380 (45)	205 (34)
	No	835 (58)	445 (53)	390 (64)
	Missing	27 (2)	13 (2)	14 (2)
One or more hospitalization				
	Yes	56 (4)	36 (4)	20 (3)
	No	1391 (96)	802 (96)	589 (97)
Inpatient Cost				
	No Cost for Visit	20 (36)	13 (36)	7 (35)
	≤5000 KS	21 (38)	13 (36)	8 (40)
	> 5000 KS	15 (27)	10 (28)	5 (25)
Any Outpatient Visit past 12 months				
	Yes	499 (34)	327 (39)	172 (28)
	No	948 (66)	511 (61)	437 (72)
Outpatient Cost				
	No Cost for Visit	154 (31)	110 (34)	44 (26)
	≤200 KS	122 (24)	67 (20)	55 (32)
	> 200 KS	223 (45)	150 (46)	73 (42)
Ever go to Herbalist				
	Yes	271 (19)	166 (20)	105 (17)
	No	1176 (81)	672 (80)	504 (83)
Herbal Cost				
	No Cost for Visit	136 (50)	90 (54)	46 (44)
	≤200 KS	69 (25)	33 (20)	36 (34)
	> 200 KS	66 (4)	43 (26)	23 (22)
Any Prescription				
	Yes	538 (37)	354 (42)	184 (30)
	No	909 (63)	484 (58)	425 (70)
Prescription Cost				
	No Cost for Prescription	191 (36)	134 (38)	57 (31)
	≤200 KS	65 (12)	42 (12)	23 (13)
	> 200 KS	282 (52)	178 (50)	104 (57)

Costs are presented in Kenyan Shillings (KS) and presented only for individuals that reported utilizing that health resource. Continuous variables are presented as “median (IQR)” and categorical variables as N (%). Percentages are by column

### Classes of healthcare utilization and costs

LCA showed optimal AIC with a three-class model (Supplemental Table 1). Details of the three classes used in the LCA are shown in Supplemental Table 2. Secondary LCA without the variable related to being told about high blood pressure produced nearly indistinguishable results to the primary LCA (Supplemental Figure 1).

**Fig. 2:**
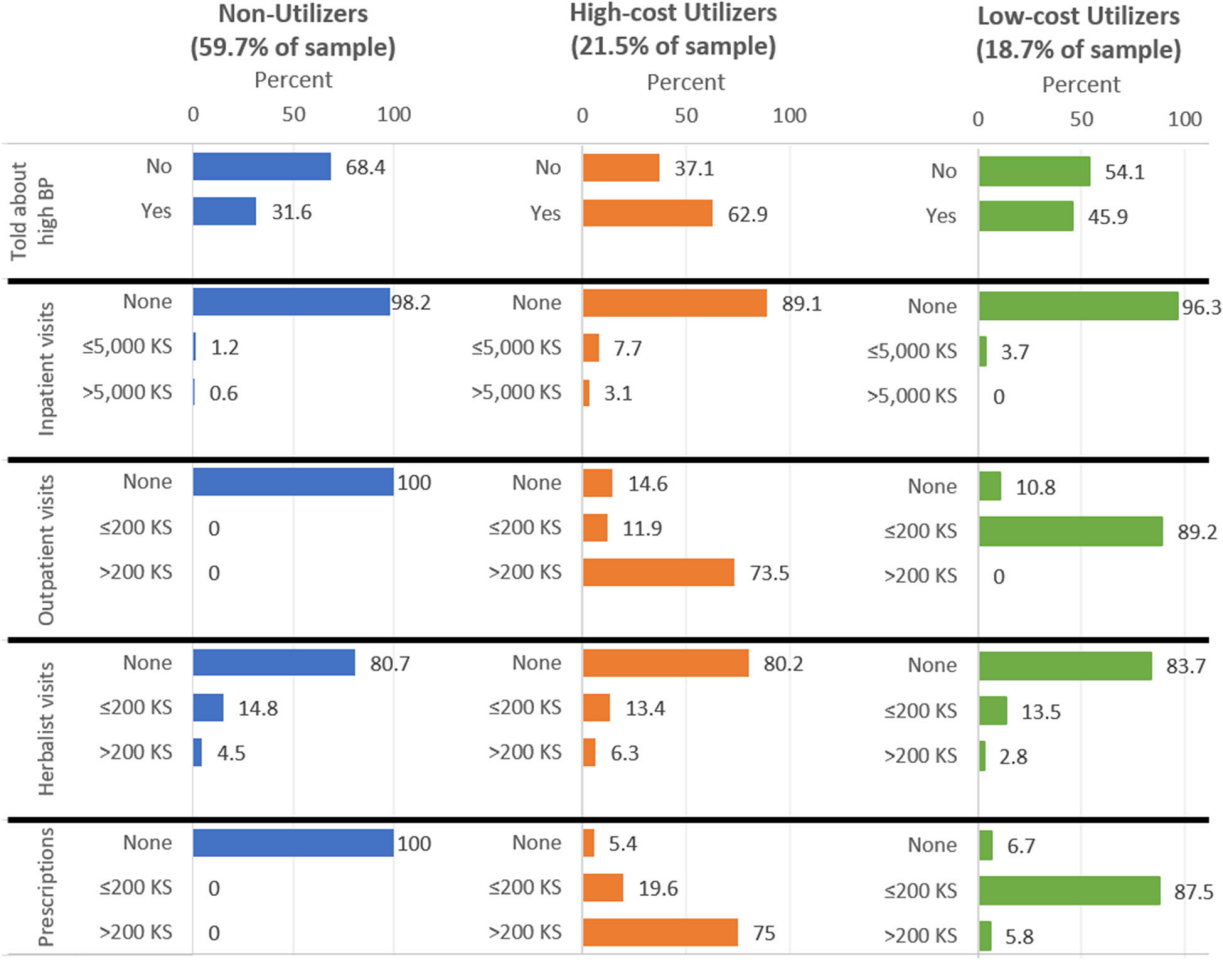
Estimated distribution of manifest (dependent) variables by latent class. Bar graphs showing manifest variable distributions across different utilization and cost parameters in each of the three LCA classes

The largest class, “non-utilizers”, comprised 60% of the population and had little to no health utilization outside of herbalist and spiritual healers (Fig. 2, Table 3). The next largest class, characterized as “high-cost utilizers”, comprised 21% of the population and reported engagement with the medical system with high cost of care (with outpatient bills and prescriptions > 200 KS (~ 2 USD)). The smallest class, characterized as “low-cost utilizers”, comprised 19% of the population and reported engagement with the medical system through outpatient visits and prescriptions with low cost of care (no outpatient bills and few prescriptions > 200 KS (~ 2 USD)).

**Table 3:** Description of health status variables stratified by the 3 latent classes

Category	Value	Total *N* = 1339	Non-utilizers *N* = 821	High-cost utilizers *N* = 279	Low-cost utilizers *N* = 256
How would you describe your mobility?					
	No problems in walking	909 (68)	592 (74)	163 (58)	154 (60)
	Some problems in walking	424 (32)	209 (26)	113 (41)	102 (40)
	Confined to bed	3 (0)	0 (0)	3 (1)	0 (0)
	Missing	3 (0)	3 (0)	0 (0)	0 (0)
How would you describe your self-care?					
	No problems with self-care	1236 (92)	757 (94)	250 (90)	229 (89)
	Some problems washing or dressing	96 (7)	45 (6)	27 (10)	24 (9)
	Unable to wash or dress myself	6 (0)	1 (0)	2 (1)	3 (1)
	Missing	1 (0)	1 (0)	0 (0)	0 (0)
How would you describe your usual activities?					
	No problems with usual activity	1068 (80)	682 (85)	197 (71)	189 (74)
	Some problems performing usual activity	250 (19)	112 (14)	73 (26)	65 (25)
	Unable to perform usual activity	18 (1)	7 (1)	9 (3)	2 (1)
	Missing	3 (0)	3 (0)	0 (0)	0 (0)
How would you describe your pain?					
	No pain	685 (51)	448 (56)	115 (41)	122 (48)
	Moderate pain	630 (47)	348 (43)	156 (56)	126 (49)
	Extreme pain	24 (2)	8 (1)	8 (3)	8 (3)
How would you describe your anxiety or depression?					
	Not anxious	599 (45)	357 (44)	131 (47)	111 (43)
	Moderately anxious	633 (47)	395 (49)	114 (41)	124 (48)
	Extremely anxious or depressed	107 (8)	52 (6)	34 (12)	21 (8)
How is your health today, 0–100?					
		75.0 (60.0, 80.0)	75.0 (65.0, 85.0)	70.0 (60.0, 80.0)	70.0 (60.0, 80.0)
	Missing	1	1	0	0

Non-utilizers had the largest proportion of men (47%) and high cost-utilizers had the largest proportion of women (67%) (Supplemental Table 2). High-cost utilizers were disproportionately younger, with 42% of the group less than the age of 50 years. Income distribution was similar across the three classes. Interestingly, high-cost utilizers had the highest rate of enrollment national insurance at 19%.

**Fig. 3:**
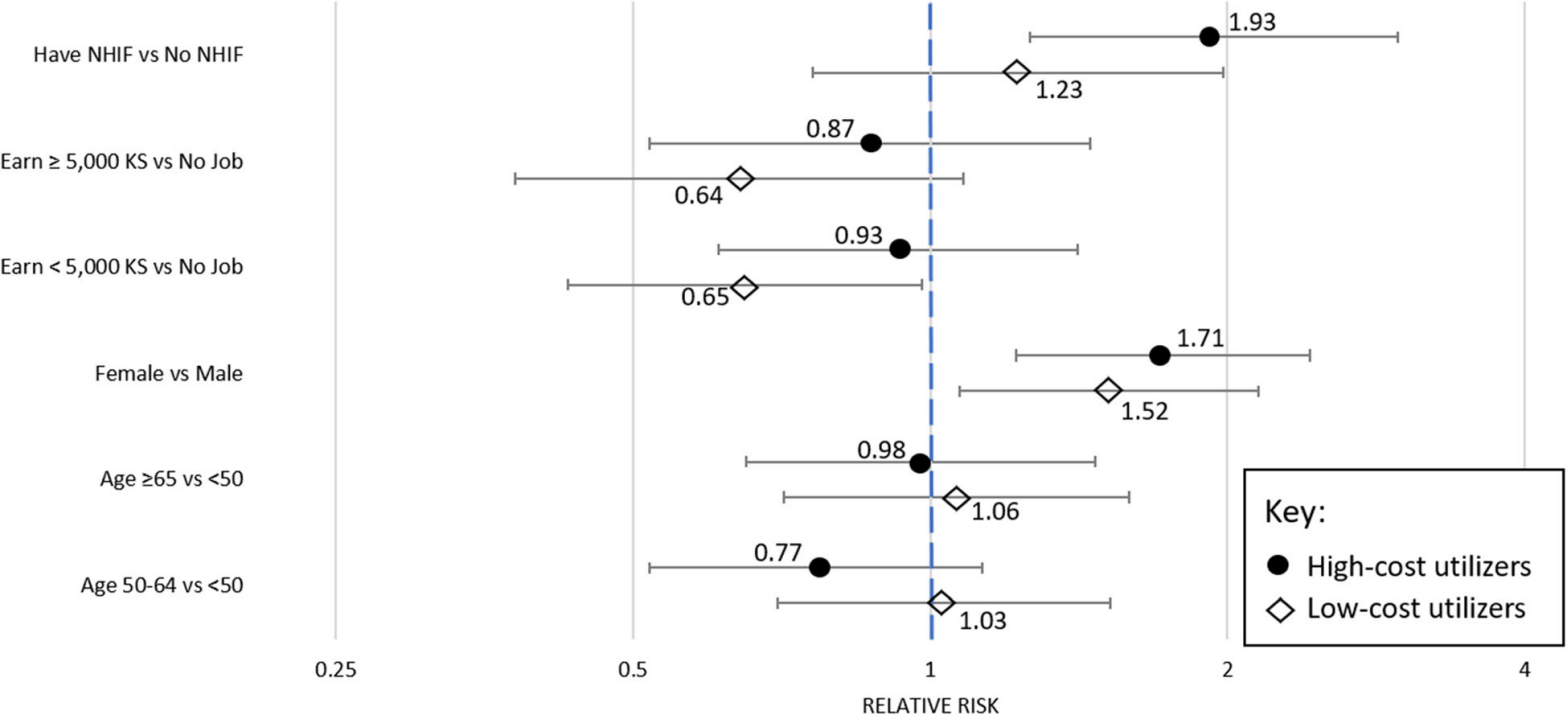
Relative risk of latent class membership by demographic*.* Relative risk of latent class membership probability compared to the largest, non-utilizer class. Error bars show 95% confidence intervals. Black dots indicate high-cost utilizer class compared to non-utilizers. White diamonds indicate low-cost utilizer class compared to non-utilizers. Please note the x-axis is logarithmic base 2. Numeric values can be found in supplement Table 3

Relative risk calculations showed sex and insurance had the strongest effect on membership in a healthcare utilization class: Women had 1.71 (95% CI: 1.22 to 2.42) times the odds of being in the high-cost utilizer class versus the non-utilization class compared to men, and 1.52 (95% CI: 1.07 to 2.15) times the odds of being in the low-cost utilizer class. Having national insurance was significantly associated with membership in the high-cost utilizer class with an odds ratio of 1.93 (95% CI: 1.26 to 2.97) (Fig. 3, Supplemental Table 3).

### Self-reported health status

The high-cost utilizer and low-cost utilizer class had similar higher rates of participants reporting difficulty with mobility, self-care, completing usual activities, and lower overall health score compared to the non-utilizer class (Table 4). The high-cost utilizer class had the highest rate of participants reporting pain*.* Overall, membership in the high-cost utilizer class was associated with a worse self-reported health (difference: − 4.03) and more problems with pain (OR 1.70), mobility (OR 1.97), self-care (OR 2.09), and usual activities (OR 2.57). Similarly, being in the low-cost utilizer class was associated with worse self-reported health (difference: − 4.55) and more problems with mobility (OR 1.69), self-care (OR 1.81), and usual activities (OR 1.79) than membership in the non-utilizer class (Table 4).

**Table 4:** Regressions of health status on health utilization latent class membership, gender, and other covariates

	Self-Reported Health (Diff, 95% CI)	Pain (OR, CI)	Anxiety and Depression (OR, CI)	Mobility (OR, CI)	Self Care (OR, CI)	Usual Activities (OR, CI)
**High-cost utilizer vs Non-utilizer**	−4.03 (−5.93, −2.17)	1.70 (1.25, 2.31)	1.67 (0.99, 2.80)	1.97 (1.43, 2.72)	2.09 (1.22, 3.57)	2.57 (1.80, 3.68)
**Low-cost utilizer vs Non-utilizer**	−4.55 (−6.52, − 2.61)	1.20 (0.88, 1.65)	1.30 (0.72, 2.35)	1.69 (1.21, 2.36)	1.81 (1.04, 3.14)	1.79 (1.22, 2.62)
**Female vs Male**	−1.49 (−2.99, 0.02)	2.04 (1.60, 2.61)	1.78 (1.11, 2.85)	1.74 (1.33, 2.28)	1.16 (0.72, 1.86)	1.67 (1.21, 2.31)
**Age 50–64 vs < 50**	−2.16 (−3.82, −0.50)	1.89 (1.44, 2.49)	1.09 (0.66, 1.79)	1.72 (1.26, 2.33)	2.73 (1.39, 5.38)	1.94 (1.33, 2.83)
**Age ≥ 65 vs < 50**	−6.03 (−7.85, −4.21)	3.05 (2.25, 4.13)	0.93 (0.54, 1.62)	3.19 (2.31, 4.41)	5.15 (2.70, 9.83)	3.19 (2.19, 4.67)
**Have NHIF vs Not**	1.61 (−0.39, 3.64)	1.44 (1.03, 2.00)	0.81 (0.42, 1.56)	0.87 (0.60, 1.25)	0.63 (0.30, 1.32)	0.85 (0.55, 1.31)
**Earn < 5000 KS vs No Job**	1.63 (−0.28, 3.53)	0.82 (0.60, 1.12)	1.74 (0.97, 3.12)	0.75 (0.54, 1.03)	0.33 (0.20, 0.54)	0.41 (0.29, 0.58)
**Earn ≥ 5000 KS vs No Job**	5.02 (2.72, 7.32)	0.55 (0.38, 0.80)	0.88 (0.42, 1.85)	0.50 (0.33, 0.76)	0.38 (0.19, 0.75)	0.35 (0.22, 0.56)

Values presented as Odds Ratio or Difference (95% confidence interval). Overall health is a score of 0–100, where higher values indicate better health. A negative effect means that women have lower reported health than men. The other symptom measures compare having any symptoms to no symptoms

Even after accounting for latent class membership probability, being a woman was associated with worse self-reported health (difference: − 1.49) and more problems with pain (OR 2.04), anxiety/depression (OR 1.78), mobility (OR 1.74), and performing usual activities (OR 1.67). Other demographic variables also were associated with self-reported health status. Older age was associated with worse health score, pain, mobility, self-care, and ability to perform usual activities with strengthened associations for the oldest age group. Similarly, earning no income was associated with worsened pain, anxiety and depression, mobility, self-care, and ability to perform usual activities than those earning greater than 5000KS. Having health insurance was associated with increased reported pain.

## Discussion

Our analysis of 1447 adults with elevated blood pressure in rural Kenya revealed that women were of poorer socio-economic status, had poorer self-reported health status, and greater healthcare utilization of outpatient visits and medication prescriptions compared to men. Three distinct patterns emerged among the entire study cohort: health care utilizers with high medical costs, health care utilizers with low medical costs, and non-utilizers. Being female and having insurance had the most influence on being in a health-utilizing class. However, across all classes, women experienced worse functional health status than men.

Our finding of greater health care utilization by women is consistent with previous reports from Kenya [20], as well as other parts of the world [3, 4, 6, 21–23]. Women overall reported worse functional health status, possibly contributing to a higher perceived healthcare need as shown in other study populations [9, 24, 25]. However, there were some notable differences and patterns that were illuminated by our latent class analysis. First, individuals with no or low utilization of health care services also had lower awareness of their elevated blood pressure. However, one-third to one-half of these individuals did endorse knowing about their elevated blood pressure, yet did not utilize healthcare. These findings are consistent with literature from other parts of the world that have described gaps in the hypertension care cascade [26, 27]. It is also possible that those non-utilizers who were aware of their elevated blood pressure faced competing obligations, such as concern about work and employment, which constrained health care-seeking behavior. Finally, contrary to what has been reported in other populations [4, 28, 29], our latent class analysis indicated that the level of healthcare utilization was similar across incomes of those employed. This unexpected finding merits further inquiry, and research is needed to clarify the factors that may impact health care utilization.

Our latent class analysis revealed one group of individuals who face higher health costs while having lower incomes. This combination of low income and high health costs is clearly concerning and highlights the urgent need for financial risk protection such as health insurance. Notably, the rates of national insurance (NHIF) enrollment among our participants was very low, with only 13% of women and 17% of men reporting current enrollment, in line with national statistics [30]. While we found that those with the highest healthcare costs had the highest rates of enrollment in NHIF, we were not able to determine whether the NHIF enrollment was initiated before or after the high-cost health care experience.

Additionally, it is worth noting that NHIF does not cover the cost of visits to herbalists or spiritual healers, seen by a substantial proportion of participants in our study, thus increasing the out-of-pocket burden for those individuals. In addition, efforts to medically engage this population need to consider collaborating with these practitioners, to maximize the reach across different segments of the population. Partnering with nontraditional medical providers in communities has been shown to be beneficial with respect to building trust and improving blood pressure control [31–33].

Kenya is considered a lower middle-income country with a 40% national unemployment rate and 36.1% of the country living under the international poverty line ($1.90/day, ~ 5700 KS/month) [34–36]. In our rural, agricultural participant population, reported unemployment rates were lower than the national average, about 21%, but almost 70% of our participants lived under the poverty line. Women felt this burden unequally, with lower rates of employment and income than men. These economic challenges have been documented in numerous countries worldwide [37]. These factors may contribute to previously studied differences in health seeking behaviors between rural and urban populations [38, 39].

Several potential strategies to improve the implementation gap with respect to blood pressure treatment and control arise from our findings. These include the need to improve community awareness of hypertension, address poverty and other social determinants of health, reduce out-of-pocket health care expenditures, and consider alternative sites of health care delivery. Community health workers can improve awareness and help to serve as a critical link between communities and the health sector [40]. Efforts to combine economic and financial programs with health care delivery are underway and actively being evaluated [41, 42]. Kenya, along with many other countries, is expanding universal health coverage in alignment with population health initiatives [43]. Finally, shifting clinical care out of the clinic and into community settings is gaining popularity and support throughout the world [32, 33, 44]. Across all of these strategies, accounting for sex-specific differences, preferences, and patterns will be critical to ensure population-level success.

We acknowledge the following limitations in our study. First, the LARK study did not collect any data on individuals who did not consent to participate in the study. We are therefore unable to assess for any differences between participants and non-participants. In addition, we did not evaluate perceptions of quality of care, and it has recently been shown that perception of quality of care can impact care-seeking behavior [45]. The sex of our participants was gathered from clinical data that were linked to the research database instead of being directly reported to the research team. In addition, all data regarding health care utilization, health care costs, and functional status were cross-sectional and self-reported and therefore subject to recall bias. We did not gather information on family income level, and it is quite likely that family members pool financial resources. Similarly, we did not collect data on education level. Lastly, the participants in the study are from rural, agricultural areas, and might not be fully representative of the general population. However, the economic challenges experienced by our study participants are not dissimilar from a large proportion of the global population. In addition, we feel that our analyses contribute to the growing literature on these issues in low-resource settings worldwide.

## Conclusions

Overall, our study found that women face unequal socioeconomic and health status compared to men with elevated blood pressure in rural western Kenya. Our findings reaffirm the need to identify population-specific barriers to seeking healthcare and develop interventions and strategies that might be sex-specific. While our study focuses on the geography of western Kenya, we believe that the findings can be relevant for low-resource, rural settings worldwide.

## Supplementary Information

**Additional file 1: Supplemental Table 1.** Comparison of different LCA models with different latent classes based on model selection statistics.

**Additional file 2: Supplemental Table 2.** Description of demographics in each latent class.

**Additional file 3: Supplemental Table 3.** Relative risk of latent class membership probability compared to the largest, non-utilizer class.

**Additional file 4: Supplemental Figure 1.** Estimated distribution of manifest (dependent) variables by latent class for secondary analysis after removing “being told about high blood pressure” manifest variable. Bar graphs showing manifest variable distributions across different utilization and cost parameters in each of the three LCA classes in the secondary analysis.

## Data Availability

The datasets used and/or analyzed during the current study are available from the corresponding author on reasonable request.

## Abbreviations

AMPATH: Academic Model Providing Access to Healthcare Partnership
SBP: Systolic blood pressure
DBP: Diastolic blood pressure
KS: Kenyan shilling – local Kenyan currency
NHIF: National Hospital Insurance Fund – Kenyan national health insurance
IQR: Interquartile range
LCA: Latent class regression analysis
USD: United States dollars
AIC: Akaike information criterion
OR: Odds ratio
CI: Confidence interval
