# Supplementary material for: Sex differences in health status, healthcare utilization, and costs among individuals with elevated blood pressure: the LARK study from Western Kenya
Source: BMC Public Health. 2021 May 19;21:948. doi: 10.1186/s12889-021-10995-3 (PMC8136119; doi:10.1186/s12889-021-10995-3)
Supplement: Supplementary file 1 — Additional file 1: Supplemental Table 1. Comparison of different LCA models with different latent classes based on model selection statistics. [file 12889_2021_10995_MOESM1_ESM.docx]

*Supplemental Table 1:* Comparison of different LCA models with different latent classes based on model selection statistics

|  | **AIC** | **BIC** |
| --- | --- | --- |
| **All** |  |  |
| 2 classes | 7553.4 | 7802.0 |
| 3 classes | 7360.9 | 7813.2 |
| 4 classes (there were problems with convergence) | 9879.4 | 10617.8 |
