# Supplementary material for: Sex differences in health status, healthcare utilization, and costs among individuals with elevated blood pressure: the LARK study from Western Kenya
Source: BMC Public Health. 2021 May 19;21:948. doi: 10.1186/s12889-021-10995-3 (PMC8136119; doi:10.1186/s12889-021-10995-3)
Supplement: Supplementary file 2 — Additional file 2: Supplemental Table 2. Description of demographics in each latent class. [file 12889_2021_10995_MOESM2_ESM.docx]

## Supplemental Table 2: Description of demographics in each latent class

| Category | Value | Total N=1339 | Non-utilizers N=804 | High-cost utilizers N=279 | Low-cost utilizers N=256 |
| --- | --- | --- | --- | --- | --- |
| Gender |  |  |  |  |  |
|  | Female | 778 (58) | 427 (53) | 187 (67) | 164 (64) |
|  | Male | 561 (42) | 377 (47) | 92 (33) | 92 (36) |
| Age Group |  |  |  |  |  |
|  | <50 | 518 (39) | 310 (39) | 117 (42) | 91 (36) |
|  | 50-64 | 436 (33) | 268 (33) | 81 (29) | 87 (34) |
|  | ≥65 | 385 (29) | 226 (28) | 81 (29) | 78 (30) |
| Monthly earnings |  |  |  |  |  |
|  | No Job | 293 (22) | 163 (20) | 58 (21) | 72 (28) |
|  | <5,000 KS | 708 (53) | 426 (53) | 154 (55) | 128 (50) |
|  | ≥5,000 KS | 338 (25) | 215 (27) | 67 (24) | 56 (22) |
| Have NHIF |  |  |  |  |  |
|  | No | 1140 (85) | 695 (86) | 226 (81) | 219 (86) |
|  | Yes | 199 (15) | 109 (14) | 53 (19) | 37 (14) |
