# Supplementary material for: Sex differences in health status, healthcare utilization, and costs among individuals with elevated blood pressure: the LARK study from Western Kenya
Source: BMC Public Health. 2021 May 19;21:948. doi: 10.1186/s12889-021-10995-3 (PMC8136119; doi:10.1186/s12889-021-10995-3)
Supplement: Supplementary file 3 — Additional file 3: Supplemental Table 3. Relative risk of latent class membership probability compared to the largest, non-utilizer class. [file 12889_2021_10995_MOESM3_ESM.docx]

*Supplemental table 3*. Relative risk of latent class membership probability compared to the largest, non-utilizer class.

|  | Low-cost Utilizers vs Non - Utilizers | High-cost Utilizers vs Non- Utilizers |
| --- | --- | --- |
| Age 50-64 vs <50 | 1.03 (0.70, 1.52) | 0.77 (0.52, 1.13) |
| Age ≥65 vs <50 | 1.06 (0.71, 1.59) | 0.98 (0.65, 1.47) |
| Female vs Male | **1.52 (1.07, 2.15)** | **1.71 (1.22, 2.42)** |
| Earn < 5,000 KS vs No Job | **0.65 (0.43, 0.98)** | 0.93 (0.61, 1.41) |
| Earn ≥5,000 KS vs No Job | 0.64 (0.38, 1.08) | 0.87 (0.52, 1.45) |
| Have NHIF vs No NHIF | 1.23 (0.76, 1.98) | **1.93 (1.26, 2.97)** |

*95% confidence intervals shown in parentheses. Bolded values have confidence intervals that exclude 1.00.*
