# Supplementary figures and images for: Sex differences in health status, healthcare utilization, and costs among individuals with elevated blood pressure: the LARK study from Western Kenya
Source: BMC Public Health. 2021 May 19;21:948. doi: 10.1186/s12889-021-10995-3 (PMC8136119; doi:10.1186/s12889-021-10995-3)

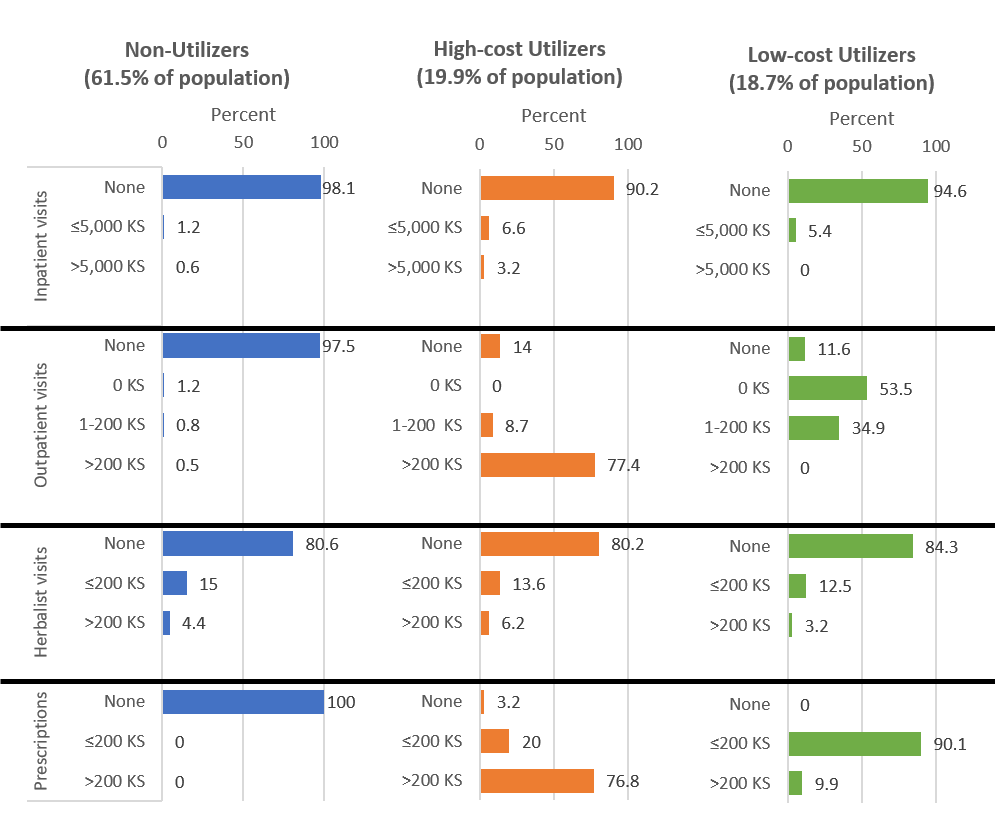

Supplement: Supplementary file 4 — Additional file 4: Supplemental Figure 1. Estimated distribution of manifest (dependent) variables by latent class for secondary analysis after removing “being told about high blood pressure” manifest variable. Bar graphs showing manifest variable distributions across different utilization and cost parameters in each of the three LCA classes in the secondary analysis. [file 12889_2021_10995_MOESM4_ESM.tif]
